# Supplementary material for: Population bottlenecks constrain host microbiome diversity and genetic variation impeding fitness
Source: PLoS Genet. 2022 May 23;18(5):e1010206. doi: 10.1371/journal.pgen.1010206 (PMC9166449; doi:10.1371/journal.pgen.1010206)
Supplement: S1 Text — (DOCX) [file pgen.1010206.s001.docx]

# S1 text

Fig. A. Fitness and nucleotide diversity of 119 lines before selection for microbiome analysis

Scatterplot of line mean egg-to-adult viability (%) and nucleotide diversity (π) as obtained through GBS of 119 lines (grey circles; from Ørsted et al. 2019 [1]). From these lines, we selected three groups of lines for analysis of the microbiome, 25 lines with low overall performance (red circles), and 25 lines with high performance (green and blue). This selection was based on a composite measure of the overall performance calculated as the sum of standardized viability and nucleotide diversity. Because we selected lines regardless of their inbred/outbred status, the ‘high’ group included nine of the outbred controls as they generally had very high fitness and genetic variation. To distinguish between the effects of genetic variation within inbred lines and population bottlenecks, we therefore differentiate between three genetic variation categories: ‘Low genetic variation’ and ‘High genetic variation’ lines and ‘Outbred’ (OB) control lines. The solid line represents the linear regression on all lines with the R^2^ value shown.


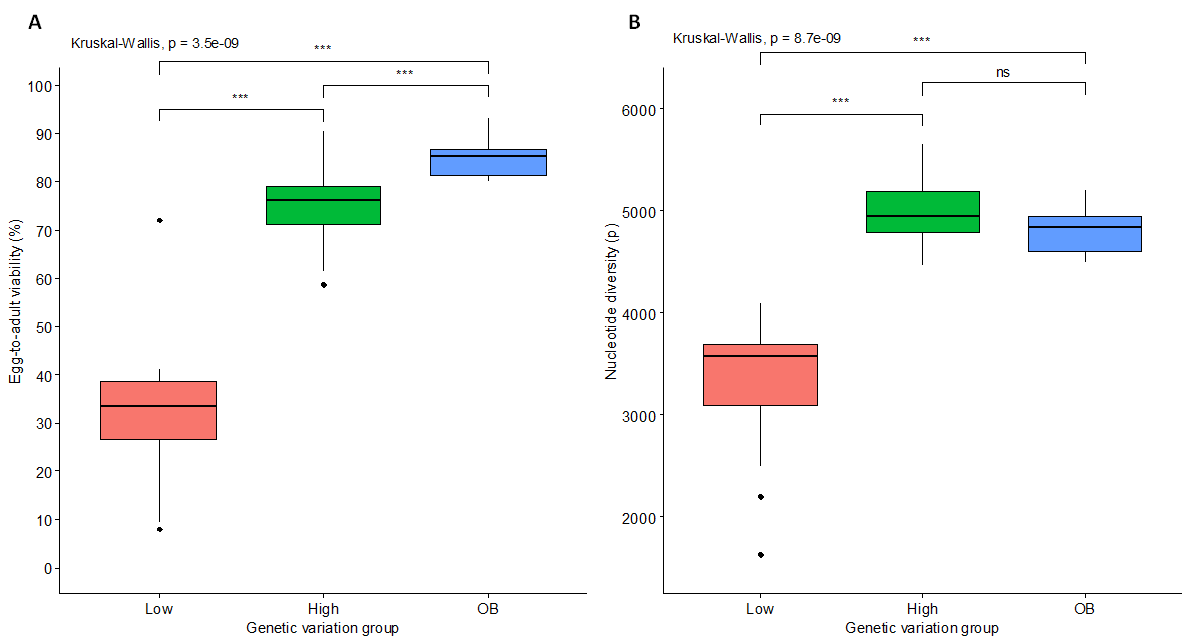
Fig. B. Egg-to-adult viability and nucleotide diversity in the three host genetic variation groups

Both the high genetic variation (‘High’) and the outbred (‘OB’) lines exhibited a significantly higher viability (**A**) and nucleotide diversity (**B**) than the low genetic variation lines (‘Low’), and viability of OB lines was higher than that of the high genetic variation lines, while nucleotide diversity was indistinguishable between these two groups (two-sample Wilcox’s t-tests; *p* < 0.05). The *p* values of a Kruskal-Wallis test show significant effects of group, while asterisks denote the results of pairwise Wilcoxon’s t-tests between groups; *** *p* < 0.001; ** *p* < 0.01; * *p* < 0.05; and ns: *p* > 0.05.


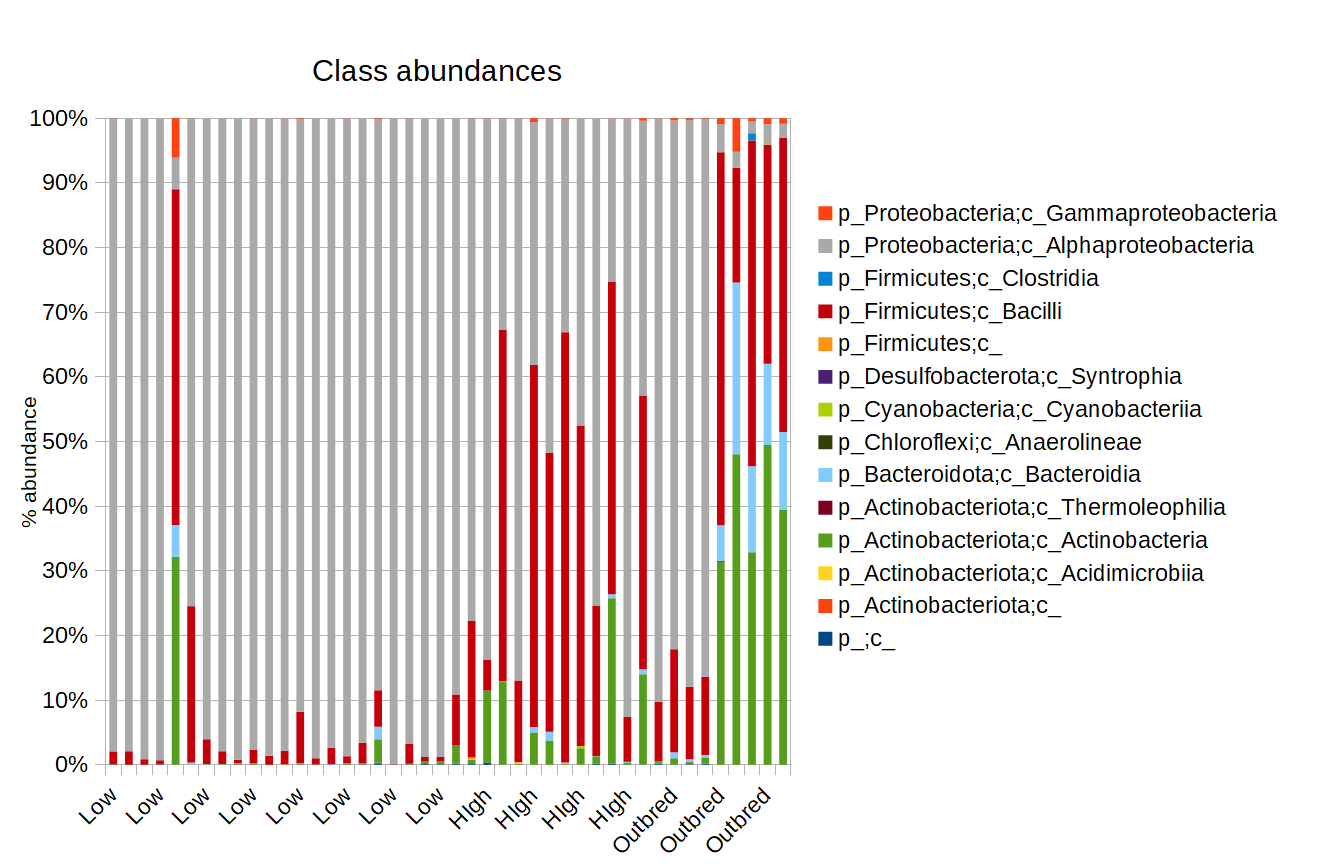
Fig. C. Taxonomic composition of host microbiomes

Bar chart of class-level taxonomic composition of the fly microbiomes in the three major groups of host genetic variation (Low: low genetic variation, High: high genetic variation, and Outbred: outbred flies).


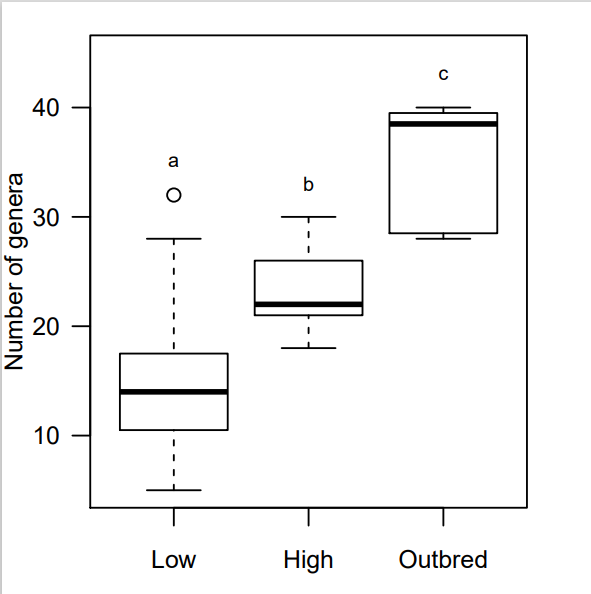


Fig. D. Number of microbial genera in the three host genetic variation groups

Boxplot of the number of bacterial genera harboured in each line, grouped according to fitness (Low: low genetic variation, High: high genetic variation, and Outbred: outbred flies). Letters denote significant differences in mean number of genera (Tukey’s HSD test, *p* < 0.05).

Fig. E. Interactions between nucleotide diversity and microbial richness on host fitness

Scatterplot of egg-to-adult viability (z-standardised) from 5 vials per line as a function of nucleotide diversity (π z-standardised) for each of the 44 lines investigated for microbial diversity; the colour of points denote observed ASV richness (alpha richness, z-standardised). The three regression lines represent regressions on lines with the highest (blue), mean (purple), and lowest (red) alpha richness, visualizing the significant positive interaction between nucleotide diversity and microbiome richness in explaining fitness of the host population (see **Table 1** in the main text for GLMM results).

Fig. F. Correlations between microbiome diversity metrics and host evolutionary capacity

Using data from Ørsted *et al.* (2019) [1] from the set of lines in the present study, we associate microbial diversity with evolutionary responses in two traits: **A.** dry body mass (in mg), and **B.** productivity (in eggs per female per day). Here evolutionary responses are defined as the slope of an ordinary linear regression across 10 generations of rearing on a stressful medium. We correlated all four measures of microbial diversity used in the present study to these two traits (eight comparisons), but here we only included the cases where there was a significant effect of microbial diversity. For both traits, we found an effect of microbial diversity (alpha richness regressed on response in dry body mass: *t*_29_ = 2.09, *p* = 0.045, and Simpson’s diversity regressed on response in productivity: *t*_29_ = 2.42, *p* = 0.022), but no effect of genetic variation, π (*t*_29_ = -1.07, *p* = 0.92, and *t*_29_ = -0.14, *p* = 0.89, for dry body mass and productivity, respectively). For details on assessment of these phenotypes, see Ørsted *et al.* (2019) [1].


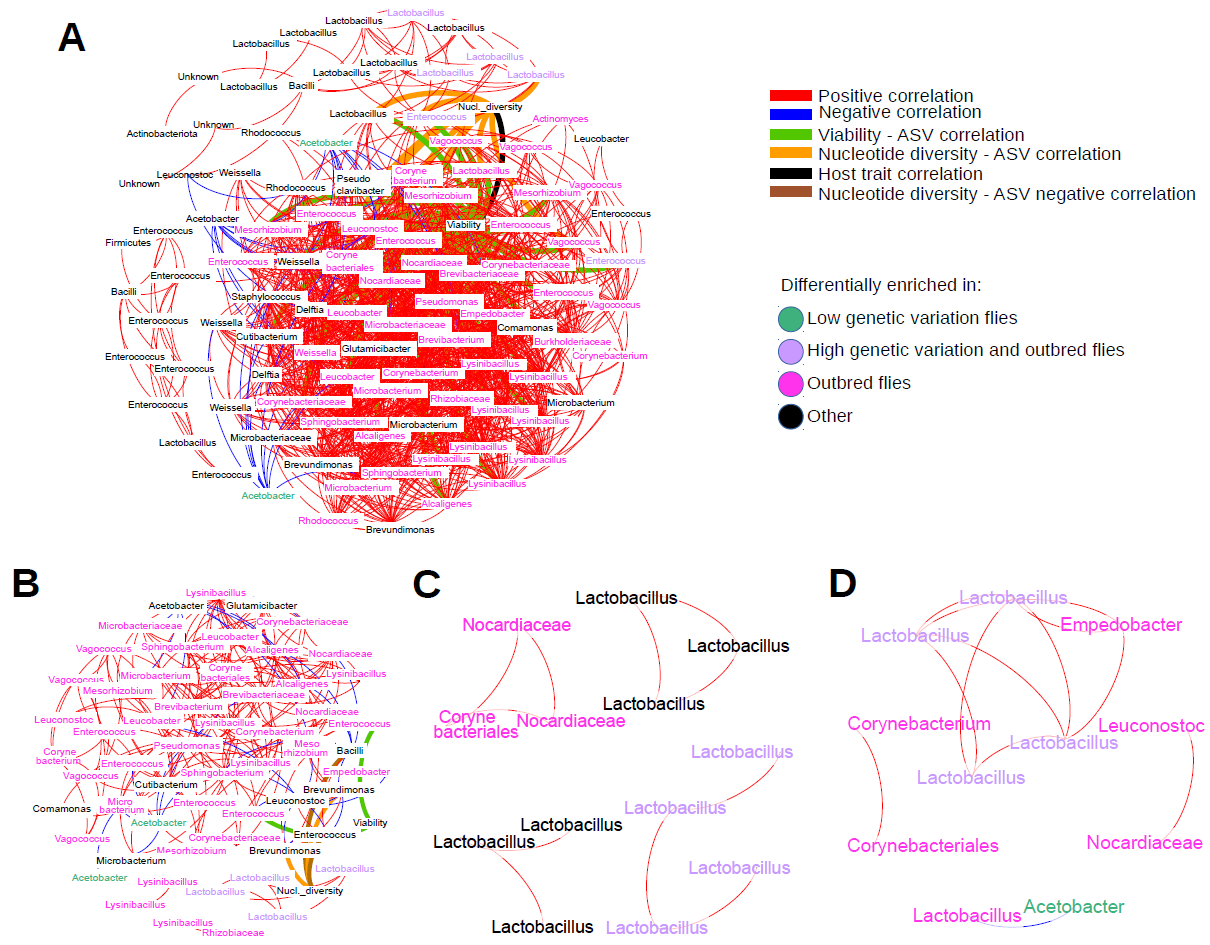


Fig. G. Microbiome co-abundance networks with ASV designations

Co-abundance networks of the fly microbiome (closely resembles **Fig. 6** in main text, except here we supply the ASVs for each node). ASVs present in at least 30 reads in total and in at least three lines, and correlations of > 0.5 (or <-0.5) and fdr-corrected *p*-values of < 0.05 are shown. The nodes are individual ASVs and the host fitness traits, egg-to-adult viability (Viability) and nucleotide diversity (Nucl. diversity), while the edges represent positive and negative correlations, and correlations linking host fitness traits and bacterial ASVs (which were positive correlations). The network containing lines from all fly groups (**A**), the outbred (**B**), high genetic variation (**C**), and low genetic variation (**D**) groups are shown with the taxonomic assignment of each node. Non-highlighted nodes that could not be taxonomically assigned are left as ASV identifier numbers. The highlighted nodes are color-coded according to the major DESeq differential abundance groups. The results of both the Leiden algorithm and degree were plotted into the network graphs as node colors and node sizes, respectively. For cross-referencing the DESeq2 differentially enriched ASVs in the networks, only the most robust differential abundance patterns were displayed, notably the ASVs that were systematically enriched in the low genetic variation flies in both the low-high and low-outbred pairwise comparisons (low), the ASVs systematically enriched in the outbred flies (outbred), and those depleted in the low genetic variation flies (*vs.* high & outbred).


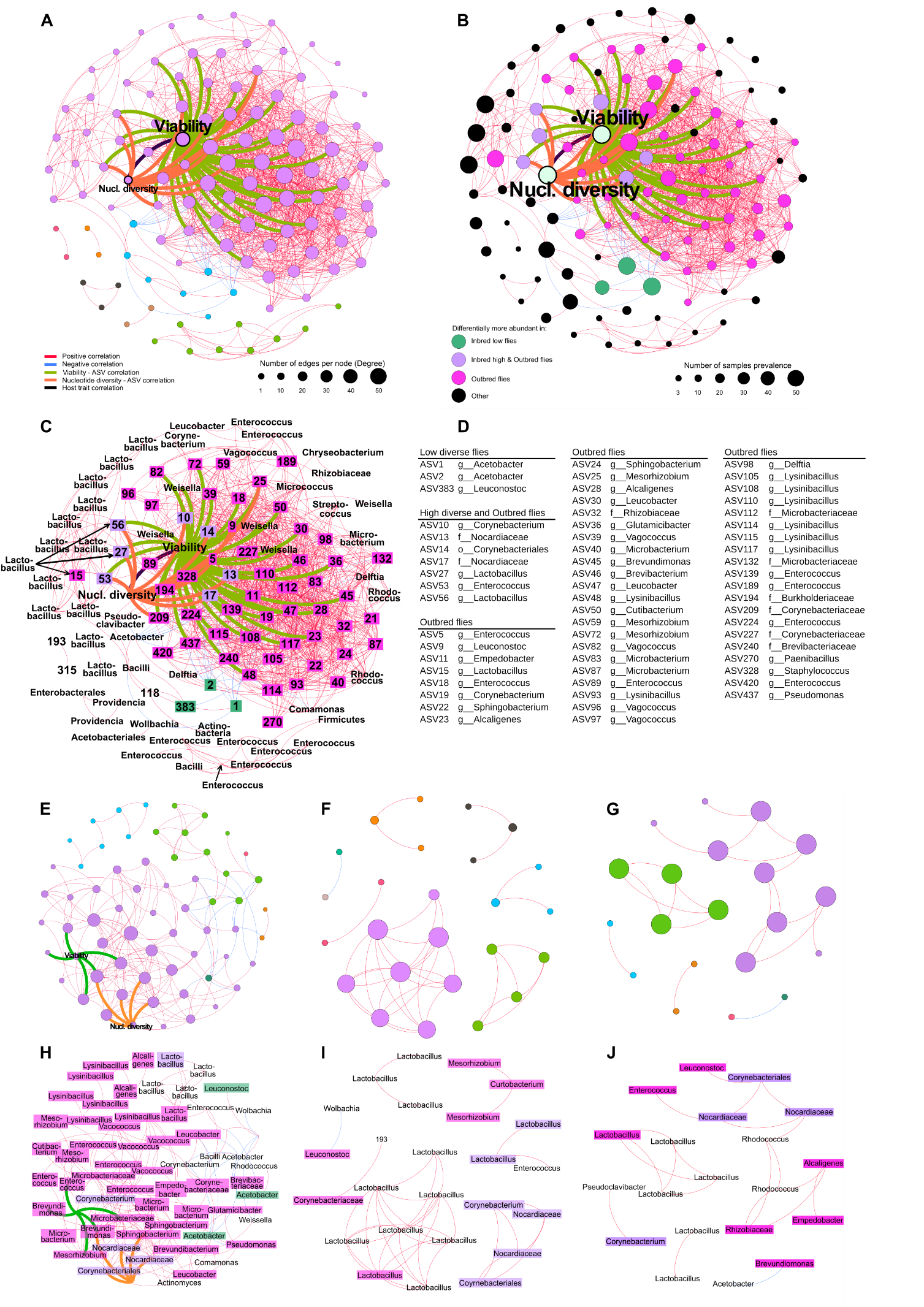


Fig. H. Microbiome co-abundance networks including removed samples with a high relative abundance of *Wolbachia*

Co-abundance networks of the fly microbiome using the ASV table that includes *Wolbachia*. ASVs present in at least 30 reads in total and in at least three lines; correlations > 0.5 (or <-0.5) and fdr-corrected p-values < 0.05 are shown. The nodes are individual ASVs and the host fitness traits, egg-to-adult viability (Viability) and nucleotide diversity (Nucl. diversity), while the edges represent positive and negative correlations, and correlations linking host fitness traits and bacterial ASVs (which were positive correlations). Panels **A, B, E, F, G** are corresponding networks found in **Fig. 6**, while **C, D, H, I, J** are corresponding networks in **Fig. G in S1 text**. The numerical values in the coloured nodes in panel **C** correspond to the ASV IDs in panel **D**. The conclusions that can be made from the dataset which included *Wolbachia* differ from conclusions made with the dataset excluding the endosymbiont. Therefore, panels **E-G** shows that reducing fly genetic variation gradually decreases microbe-microbe associations in the fly microbiome, while **Fig. 7C, 7D, and 7E** shows that fly genetic variation and microbe-microbe associations do not monotonically decrease; rather, inbred flies host microbes that do not interact much compared to outbred flies.


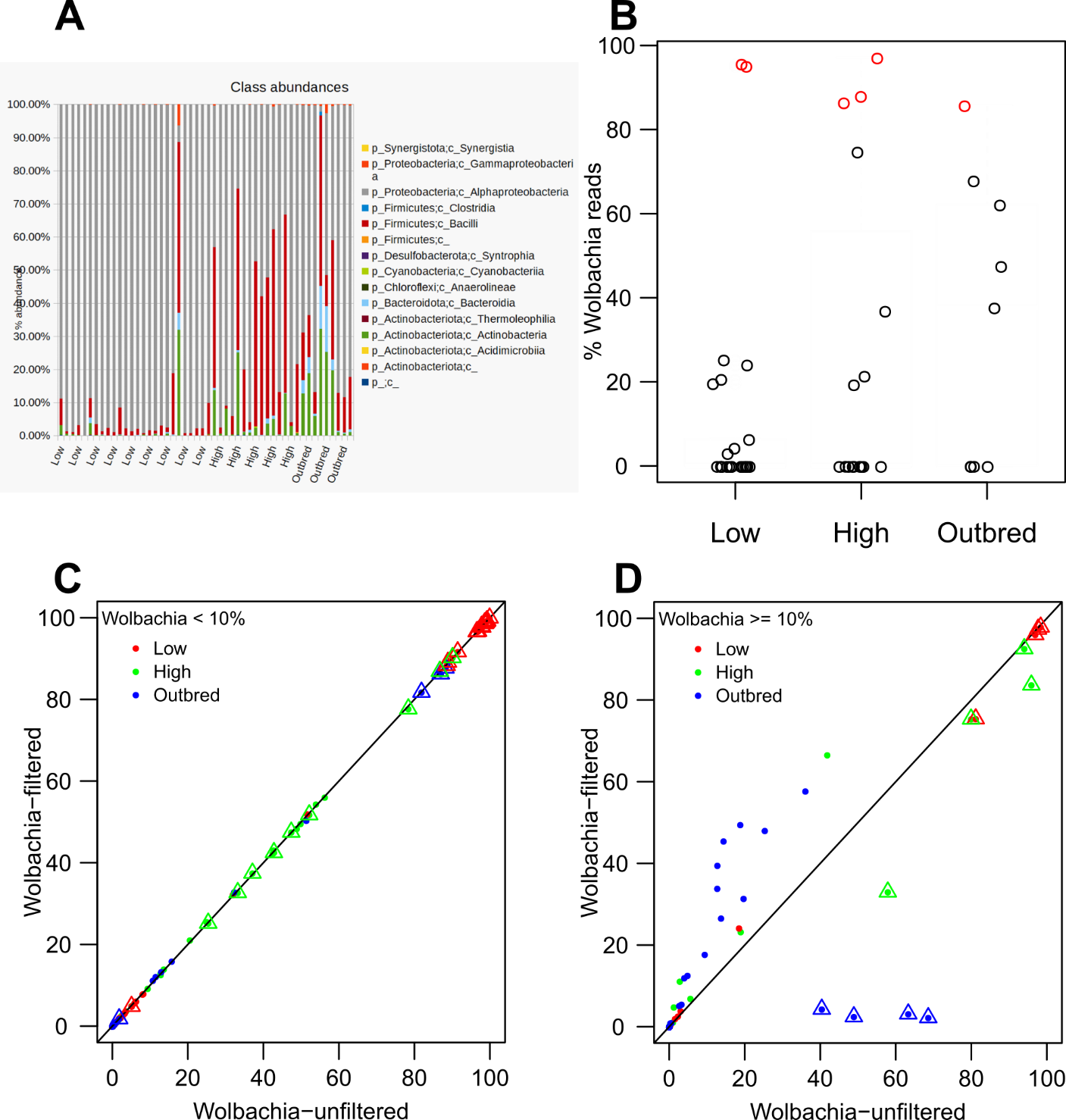


Fig. I. Effects of removing samples with a high relative abundance of *Wolbachia*

(**A**) Bar chart of class-level taxonomic composition of the fly microbiomes in the three major groups of host genetic diversity when *Wolbachia* reads are included in the frequency table. (**B**) Relative abundance of the endosymbiont *Wolbachia* ASV, across all 50 initially selected lines . We removed six lines total with abundances above a 85 % threshold (red circles; two low genetic variation, three high genetic variation and one outbred). No effect of genetic variation group on relative abundance was found (Kruskal-Wallis rank sum test; χ^2^ = 2.54, df = 2, *p* = 0.28). (**C**) The percent proportion of the bacterial class-level taxa from the lines containing less than 10% *Wolbachia* were plotted for the dataset where *Wolbachia* was included and for the dataset where *Wolbachia* was removed. (**D**) The proportion of the class-level taxa from lines with 10% or more *Wolbachia* reads were also plotted. Each dot corresponds to one class from one sample. Given that *Wolbachia* belong to the Alphaproteobacteria, the dots corresponding to Alphaproteobacteria are overlaid with triangles. The regression line indicates the plot region where taxon proportions are 1:1 ratio between the two axes. When *Wolbachia* abundance was low, the community profile was highly similar between the dataset with and without *Wolbachia*, whereas, when *Wolbachia* abundance was high, their prevalence skewed the relative proportion of the other taxonomic groups.

Table A. Results of general linear mixed models

Results of general linear mixed models (GLMMs) of egg-to-adult viability as a function of nucleotide diversity (NuclDiv) and microbiome diversity and their interaction as fixed effects. The measures of microbiome diversity are divided into alpha richness indices; estimated ASV richness (chao1), and diversity indices accounting for relative abundances as well (Shannon-Wiener index (Shannon) and Simpson’s index (Simpson) in each of their own model. Both dependent and independent variables are scaled (Z-standardization) to allow direct comparison of effect sizes. Replicate vial ID were included as a random effect, as flies from the same vial are not considered independent. Conditional coefficients of determination of the GLMMs $\left( R_{full model}^{2} \right)$ interpreted as the variance explained by the entire model, including both fixed and random effects, is shown. Asterisks denote the significance of individual variables or interactions; *** *p* < 0.001; ** *p* < 0.01; and * *p* < 0.05. The full model including both dependent variables and their interaction is compared with individual models with either nucleotide diversity or alpha diversity with a χ^2^ test.

Table B. Fly medium ingredients and vendors

Amount per liter and vendor of standard fly medium ingredients used in the present study

| **Ingredient** | **Amount per L** | **Vendor** |
| --- | --- | --- |
| Yeast | 60 g | Instaferm, Lallemand Inc., Canada |
| Sucrose | 40 g | Nordic Sugar, Denmark |
| Oatmeal | 30 g | Salling, Denmark |
| Agar | 16 g | PanReac AppliChem ITW Reagents, Germany |
| Nipagen (Methylparaben) | 12 mL | Sigma-Aldrich, Merck, Germany |
| Acetic acid | 1.2 mL | Sigma-Aldrich, Merck, Germany |

References

1. Ørsted M, Hoffmann AA, Sverrisdóttir E, Nielsen KL, Kristensen TN. Genomic variation predicts adaptive evolutionary responses better than population bottleneck history. PLOS Genetics. 2019;15: e1008205. doi:10.1371/journal.pgen.1008205
